# Consequences of Heat Stress on Physiology, Microbiome Dynamics, and Multi-Omics in Dairy Cows: More than Meets the Eye

**DOI:** 10.3390/biology15120918

**Published:** 2026-06-12

**Authors:** Themistoklis Giannoulis, Eleni Dovolou, Zissis Mamuris, Georgios S. Amiridis

**Affiliations:** 1 Department of Animal Science, University of Thessaly, 41223 Larissa, Greece; thgianno@uth.gr (T.G.); entovolou@uth.gr (E.D.); 2 Department of Obstetrics & Reproduction, Faculty of Veterinary Science, University of Thessaly, 43100 Karditsa, Greece; 3 Laboratory of Genetics, Comparative and Evolutionary Biology, Department of Biochemistry and Biotechnology, University of Thessaly, 41500 Larissa, Greece; zmamur@uth.gr

**Keywords:** dairy cattle, heat stress, thermotolerance, reproductive physiology, epigenomics, multi-omics, intergenerational effects, gut microbiome, mTOR signaling, rumen dysbiosis

## Abstract

Heat stress (HS) is at the top of the challenges facing modern dairy production, with annual losses according to global projections, under high-emission scenarios, reaching US$14.7–40.0 billion by the end of the century. This review emphasizes three interconnected topics that account for most of the proportion of the productive and reproductive losses during HS. First, the physiological consequences of HS are reviewed, with emphasis on the pair-fed thermal neutral (PFTN) paradigm, which established that reduced dry matter intake (DMI) accounts for only 35–50% of the observed milk yield decline, with the remainder arising from tissue-level effects of hyperthermia on mammary function, metabolism, and reproductive performance. Second, HS-induced microbiome disruption is examined as an active pathophysiological amplifier, whereby rumen dysbiosis compromises intestinal barrier integrity and drives systemic endotoxaemia, chronically amplifying the immune suppression already imposed by the thermal insult. Third, we focus on the integration of multi-omics platforms as a management approach, since single-omics analyses capture only a fraction of the biological complexity underlying the HS response. As the available datasets expand in coverage and scale, their integration through AI-driven analytical frameworks has the potential to substantially advance beyond the current fragmented picture, progressively building toward a systems-level model of thermal stress. Evidence-based mitigation strategies spanning environmental cooling, targeted nutritional supplementation, and genomic selection are critically evaluated within this framework, with emphasis on equity of access to evidence-based solutions across global dairy production systems.

## Simple Summary

Heat stress is one of the foremost challenges facing modern dairy production, with projected global economic losses reaching tens of billions of dollars by the end of the century under high-emission scenarios. This review addresses three interconnected dimensions of its pathophysiology that collectively account for the majority of productive and reproductive losses. First, the physiological consequences of hyperthermia are examined, with emphasis on the pair-feeding paradigm, which establishes that reduced feed intake explains only 35–50% of milk yield decline, with the remainder arising from direct tissue-level effects of heat on mammary function, metabolism, and reproductive performance. Second, heat stress-induced microbiome disruption is presented as an active pathophysiological amplifier, whereby rumen dysbiosis compromises intestinal barrier integrity and drives systemic endotoxaemia, chronically amplifying immune suppression. Third, the integration of multi-omics platforms is highlighted as essential for capturing the full biological complexity of the heat stress response, given that single-omics analyses provide only a partial picture. As available datasets expand and AI-driven analytical frameworks mature, a systems-level understanding of thermal stress will emerge—one capable of informing evidence-based mitigation strategies spanning environmental cooling, targeted nutritional supplementation, and genomic selection for thermotolerance.

## 1. Introduction

Global mean surface temperature has risen by approximately 1.1 °C above pre-industrial levels as a direct consequence of anthropogenic greenhouse gas emissions, and the worst-case scenario, with high-emission trajectories, projects an additional 3.3–5.7 °C of warming by 2100 [1]. For the dairy cattle sector in the Mediterranean basin, the Middle East, and tropical regions, this scenario represents a reality that requires imminent action to secure the sustainability of the sector, according to the targets of the European Green Deal. HS is already the largest single environmental threat to dairy production globally, with annual economic losses in the United States alone estimated at US$2.9 billion [2], and end-century global projections under high-emission scenarios that justify urgent interdisciplinary action [3]. In Europe, 11–21.6% of cattle are projected to experience at least 15 additional heat-wave days by 2050, with naturally ventilated indoor systems more exposed to sustained thermal entrapment than outdoor grazing systems [4]. These numbers demand interdisciplinary cooperation that could lead to a systems-level understanding of HS, which will become the basis of the development of novel practices for the mitigation of the adverse effects of heat stress. A century of research into stress physiology has established the foundational mechanisms of HS [5,6]; yet, the pace of climate change [7] and its disproportionate impact on temperate dairy systems [8] are generating challenges that existing single-domain frameworks are insufficient to address. The capacity of animals to adapt to thermal challenge is limited and a long-lasting process, and is affected by factors such as the species, the breed and the production level [9], pointing out the need for integrative mitigation strategies that span environmental management, genetics/genomics, and nutrition.

The high-yielding Holstein cow represents a very good example for the study of negative effects of HS and the design of management practices to mitigate the effects and secure the viability of the dairy sector. Decades of intensive genetic selection for milk production have maximized productive potential and decreased thermal resilience: the negative genetic correlation between milk yield and the temperature–humidity index (THI) threshold for productive decline (r ≈ −0.53) is a direct consequence of the metabolic heat generated by supporting lactation output [10,11]. High-producing cows generate substantially more body heat than their low-producing counterparts, and the resulting erosion of thermal tolerance has made modern genetics more difficult to respond to in a warming climate.

Furthermore, for decades, HS-induced milk yield losses were attributed almost entirely to reduced DMI, and management responses were designed accordingly [5,6]. A recent meta-analysis quantified that heat stress reduces feed intake by 10–20%, milk yield by 10–25%, and feed efficiency by up to 15% across diverse production systems [12], with lactational performance impacts documented as a consistent multi-trait phenotype [13]. Daily patterns of heat production under thermoneutral conditions confirm that high-yielding cows generate metabolic heat at rates that exceed their dissipation capacity even before ambient temperatures rise [14], and heat stress further suppresses fat content in milk through disrupted de novo lipogenesis [15]. The PFTN paradigm established that 50–65% of productive losses arise from direct hyperthermia-driven tissue effects independent of DMI [16,17,18], meaning that strategies targeting DMI alone address only half the problem.

Nowadays, the gastrointestinal microbiome is increasingly recognized as an active player of HS-induced pathology: compositional shifts in rumen and hindgut microbial communities reduce short-chain fatty acids (SCFA) output, while intestinal ischemia—a consequence of blood flow redistribution toward peripheral skin vessels for evaporative cooling—compromises epithelial tight junction integrity, enabling lipopolysaccharides (LPS) translocation and chronic systemic low-grade endotoxemia through TLR4/NF-κB activation [19,20,21]. The molecular multi-omics architecture of the HS response is a second underexplored topic, and the development of high-throughput sequencing techniques and AI-driven algorithms, coupled with the evolution of genomic selection strategies that leverage molecular insights to enhance prediction accuracy, has enabled the integration of multi-omic data. This synergy provides a comprehensive understanding of the molecular changes occurring during heat stress, ultimately facilitating the design of advanced breeding programs aimed at optimizing performance. By integrating physiological, microbial, and molecular evidence, this review outlines the mechanisms underlying heat-induced dysfunction and identifies biological pathways with potential to enhance the resilience of dairy cows’ productivity under hostile environmental conditions.

## 2. Thermoregulation and the Physiological Consequences of Heat Stress

### 2.1. Thermal Physiology, Thermoneutral Zone (TNZ), and Limitations of the THI

Dairy cattle are homeotherms that maintain body temperature within 38.0–39.5 °C through the coordinated regulation of endogenous metabolic heat production and heat loss [22]. Within the TNZ, which is approximately 5–25 °C for adult Holstein cows, this balance requires no additional energetic expenditure and allows dietary energy to be fully allocated towards productive and reproductive pathways [23]. Above the TNZ, cows activate coordinated thermoregulatory responses, which include increased respiratory rate, redistributing blood flow toward peripheral skin vessels, and voluntarily reducing feed intake to lower the metabolic heat. These responses are rather costly in terms of energy demands, compounding the primary impact of hyperthermia and contributing to the negative energy balance (NEB) observed during HS [22]. The physiology of acclimation involves both acute and long-term responses in neuroendocrine, metabolic, and immune function that partially attenuate the negative effects of HS without fully restoring productive capacity [24]. Adaptation strategies to high temperatures encompass phenotypic plasticity, genetic adaptation, and management-driven amelioration, and differ substantially across livestock species and production contexts [25]. Extensively managed ruminants in tropical environments have evolved a broader array of behavioral and physiological heat-mitigation strategies than intensively managed dairy breeds, yet the persistence of long-lasting high temperatures leads to productive impairments and welfare issues in these breeds as well [26].

The THI has been used for six decades as the primary tool to assess the effects of exposure to elevated environmental temperatures in dairy cows [23,27]. A THI of 68–72 is broadly accepted as the upper tolerated threshold in Holstein cows, though this threshold is lowered substantially to 60–65 for cows producing more than 35 kg/day, due to their additional internal metabolic heat generated [28,29,30]. Yet, THI has attracted methodological criticism, since it does not consider wind speed, individual animal variation in coat characteristics, body condition, and metabolic heat production [31]. An increasing concern is that nocturnal THI is rising faster than daytime THI under high-emission scenarios, progressively narrowing the overnight restoration period that allows cows to dissipate accumulated heat load [32]. Alternative indices incorporating solar radiation and wind speed, such as the Black Globe Humidity Index (BGHI), the Comprehensive Climate Index (CCI), and the heat load index (HLI), have demonstrated improved physiological correlations in specific contexts, though none has achieved universal adoption in dairy systems due to data availability constraints and limited cross-regional validation [33].

At the cellular level, temperature sensing is initiated through thermosensitive Transient Receptor Potential (TRP) channels and TRPM2, which regulate intracellular Ca^2+^ flux and process thermal signals to the hypothalamus [34,35]. TRPV4-mediated Ca^2+^ influx under elevated temperatures leads to mitochondrial Ca^2+^ overload, disrupting oxidative phosphorylation and triggering a reactive oxygen species (ROS) leak that initiates the cellular pathological cascade described in Section 2.4 [34]. The identification of polymorphisms in TRPM2 in cattle from chronically heat-exposed populations illustrates how population-level genomic adaptation to thermal environments is detected through molecular signals that could be utilized for marker-assisted thermotolerance selection [34].

### 2.2. The Central Role of Hyperthermia and mTOR Suppression in Declining Milk Production

The role of reduced feed intake in the reduced productive performance has been extensively studied by several researchers, and the results are indicative of its implications: By comparing thermoneutral and heat-stressed cows under the same DMI, these experiments showed that reduced feed intake accounts for only approximately 35% of the HS-induced decrease in milk yield [16,17,18]. The study has been replicated and extended by multiple independent researchers, and the results confirm that 50–65% of productive losses arise from direct tissue-level effects of hyperthermia independent of any change in DMI [36]. This means that the strategies focusing exclusively on maintaining DMI under HS are addressing only half of the proportion of the factors responsible for the reduced performance under HS.

At the molecular level, the effects of hyperthermia on mammary protein synthesis are mediated through suppression of mammary mTOR complex 1 (mTORC1) signaling, which acts as the main nutrient-sensing kinase that integrates amino acid availability, energy balance, and growth factor signaling to regulate protein translation. Hyperthermia downregulates mTOR, AKT1, RPS6KB1, and RPS6 mRNA expression alongside amino acid transporter genes, and the casein genes CSN1S1, CSN2, and CSN3, while inducing apoptosis through upregulation of BAX and suppression of BCL2 [37]. A comprehensive review of the influences of HS on bovine mammary gland function confirmed that direct effects of hyperthermia on mammary secretory cells are central to the observed productive decline [38]. The mTOR suppression is exacerbated by massive upregulation of HSP70 which directly competes with the mammary gland for circulating AAs [39]. The molecular alterations observed during HS extend well beyond the period of thermal exposure. Dado-Senn et al. [40] demonstrated that HS during the dry period leaves a lasting effect on the mTOR signaling network of the subsequent lactation mammary gland: cows exposed to HS during the dry period exhibited the same patterns in mammary tissue at 14, 42, and 84 days in milk, despite post-calving management under cooling conditions. This means that the molecular machinery of mTOR suppression is not recalibrated when the thermal insult ends, but it persists in altered signaling mechanisms, leading to impaired protein synthetic capacity in the subsequent lactation, a carry-over effect whose economic cost is almost overlooked in HS loss evaluations.

### 2.3. The Paradoxical HS Metabolic Phenotype

The status of NEB is a common feature for the high-producing cows in early lactation, since the consumption of dry matter cannot fulfill their energetic demands [41] for milk production, leading to the reduction in body condition score (BCS). This energetic shortfall activates a series of endocrine and metabolic changes, such as the increased secretion of GH and the decreased secretion of insulin [42], leading to lipid mobilization and raised levels of non-esterified fatty acids (NEFAs), which serve as an alternative nutrient for peripheral tissues and preserve glucose for mammary lactose synthesis. However, during HS, this is not the case during NEB: basal NEFA concentrations are reduced (by 39.8% relative to PFTN controls [18]), reflecting active inhibition of lipolysis despite the energy deficit. The occurring hyperinsulinemia drives GLUT4-mediated glucose uptake in skeletal muscle and adipose tissue, shifting the whole-body energy substrate toward glucose catabolism and away from the mammary gland, negatively affecting the production [18,35,43]. Blood glucose simultaneously declines by approximately 8% [18] because hepatic gluconeogenesis is not sufficient to cover enhanced peripheral glucose utilization.

Baumgard and Rhoads [36] interpreted this “insulin–glucose paradox” as a strategy to alleviate HS by lowering the increased metabolic heat of lipolysis and the partition of the remaining glucose toward peripheral thermoregulatory tissues. Metabolic and hormonal acclimation to HS involves progressive neuroendocrine, hepatic, and adipose tissue adjustments that deprive nutrients from productive functions, in favor of thermal survival [29]. Collier et al. [44] claimed that these shifts are a result of nutrient partitioning, with the regulatory signals redirecting energy toward thermoregulation at the expense of production. Body condition score at HS seems to affect this response, since greater BCS is associated with increased NEFA mobilization and the retention of milk yield [45], while pre-existing oxidative stress at the periparturient period exacerbates HS-induced antioxidant depletion [46]. The strategy makes sense in the context of thermal survival but is detrimental for milk production, because it halts the fat mobilization that would otherwise buffer the mammary gland through NEB. Thus, the approach of dietary strategies designed to drive propionate production and hepatic glucose supply—which may be more appropriate for heat-stressed than for thermoneutral cows—deviates directly from this understanding but has been insufficiently incorporated into practical feeding programs [47]. Thyroid hormones T3 and T4 are simultaneously reduced, lowering basal metabolic rate as an adaptive strategy [48]. The somatotropic axis is disrupted with hepatic GH resistance partially uncoupling the GH-IGF-I axis [49]. Proteomics analysis in adipose tissue in pregnant cows during HS showed an enrichment in oxidative stress response pathways [50].

### 2.4. Immune Dysfunction, Oxidative Stress, and the MAPK/NF-κB/AMPK Signaling Network

HS compromises immune competence through a series of hormonal, cellular, and transcriptomic mechanisms that are mutually reinforcing [51,52,53]. The HPA axis activation leads to cortisol hypersecretion (reported as approximately 1.8-fold after 72 h of HS in some experimental conditions), which remains even during the night, which is usually a restoration period [34]. This phenomenon is responsible for the suppression of T-cell proliferation by 40–50%, for the reduction in antibody concentrations by more than 30%, for the promotion of neutrophil apoptosis and for the downregulation of L-selectin expression, which systematically shifts adaptive immunity from TH1 toward TH2 responses [51,52]. This TH1 → TH2 shift is particularly damaging in the context of mastitis predisposition. Concurrent ROS overproduction, proposed to involve TRPV4-mediated mitochondrial Ca^2+^ overload and NADPH oxidase activation—a mechanistic axis supported by in vitro evidence though requiring further validation in ruminant models—depletes antioxidant reserves [34,54].

Three signaling cascades mediate these effects: (i) MAPK (p38/JNK) activation drives apoptosis via HSP27 nuclear translocation; (ii) ROS-mediated NF-κB activation upregulates IL-6, TNF-α, and IL-1β alongside acute-phase proteins; and (iii) AMPK activation, initiated by Ca^2+^/calmodulin-dependent pathways, partially antagonizes NF-κB through SIRT1-mediated p65 deacetylation and promotes mitochondrial biogenesis [34]. The immune dysfunction imposed by cortisol hypersecretion and ROS overproduction predisposes to infection, which in turn intensifies systemic inflammation and further impairs thermoregulatory capacity [55]. The welfare consequences of this immune-inflammatory cascade are substantial, encompassing not only mastitis but also reduced time for lying, feeding, and rumination that collectively compound productive losses [56]. Mild heat stress elicits adaptive immune responses in blood mononuclear cells and mesenteric lymph node leukocytes that become maladaptive under sustained thermal exposure [57]. Core body temperature, time of day, and ambient climate conditions interact to determine the degree of behavioral displacement in heat-stressed cows, with peak afternoon temperatures driving the most severe feeding and resting time reductions [58]. Rumination time is suppressed under HS in a pattern that reflects deterioration of metabolic status and health, and its monitoring can serve as an early non-invasive indicator of thermal compromise [59]. The clinical consequences affect multiple organ systems: HS increases mastitis incidence 2–3-fold [34], and heat stress during lactation significantly reduces circulating inflammatory markers including acute-phase proteins (serum amyloid and haptoglobin) in both systemic and mammary compartments [12]. Cows calving during hot months show elevated levels of retained fetal membrane, subclinical ketosis, displaced abomasum, and mastitis relative to those calving in cooler months [60]. Further, the incidence of uterine infections is substantially higher during summer [61,62], while HS-stressed cows spend up to 3 h less lying per day, increasing lameness risk [63,64]. In a systematic review more than 200 distinct animal-based indicators of HS resilience in dairy cattle have been identified, indicating the wide spectrum of disruptions [65]. When severe HS progresses to lethal heat stress, a cascade involving electrolyte dyshomeostasis and multiple organ failure can result in death even after environmental temperatures have normalized [66,67]. Breed-specific responses are documented, with zebu-derived breeds consistently demonstrating superior thermal resilience attributable in part to constitutively higher HSP70 expression and more effective antioxidant systems [68,69].

Beyond the well-characterized lymphocyte suppression, HS exerts equally significant effects on innate immune cell function. Neutrophil chemotaxis, oxidative burst capacity, and phagocytic activity are all reduced under hyperthermic conditions, impairing the first line of defense against bacterial pathogens at precisely the time when environmental pathogen pressure is highest [70,71]. Natural killer cell cytotoxic activity is concurrently reduced, and monocyte antigen-presenting capacity is compromised through downregulation of MHC class II expression, collectively weakening the bridge between innate and adaptive immune responses [71]. The extracellular role of HSP70 adds a further layer of immune dysregulation. While intracellular HSP70 exerts cytoprotective effects, its extracellular form acts as a danger-associated molecular pattern (DAMP), activating TLR2 and TLR4 on antigen-presenting cells and amplifying the systemic inflammatory cascade [72]. This extracellular HSP70 signaling contributes to the chronic low-grade inflammatory state characteristic of dairy cows during the summer, paradoxically coexisting with suppressed pathogen-specific immune responses. At the level of mammary defense, lactoferrin and lysozyme concentrations in milk—key components of non-specific mammary immunity—are reduced during HS, reflecting direct suppression of their synthesis in mammary epithelial cells, alongside the altered milk composition of the heat-stressed cow [38]. Finally, prolactin—which normally exerts immunostimulatory effects through PRL receptors on lymphocytes—is paradoxically elevated during mild HS but declines sharply under severe sustained hyperthermia, removing an important immunomodulatory signal at the point of greatest immune need [6].

### 2.5. Reproductive Consequences: From GnRH Suppression to the Epigenetically Scarred Offspring

#### 2.5.1. Hormonal and Endocrine Dysregulation

Reproductive performance is of great importance, since it is the main factor affecting the sector’s viability and sustainability as well as long-term productivity, and its reduction during HS should be under very careful consideration [73,74,75]. The effects of HS in reproduction span multiple levels, including hormonal regulation, follicular development, oocyte quality, embryo development, and placentation. The influence of HS on reproduction operates across multiple temporal scales, primarily originating from dysregulations of the hypothalamic–pituitary–gonadal axis [76]. This could cause shortening of the duration of the estrus expressions, multiple ovulations, delayed ovulation or even anovulation [77]. The immediate endocrine impacts include cortisol-driven suppression of GnRH leading to reduced LH surge, endometrial prostaglandin F2α hypersecretion, causing premature corpus luteum regression, reduction in plasma progesterone and direct thermal effects on hypothalamic neuronal signaling [73,78].

Bovine endometrial epithelial cells exposed to heat stress in vitro exhibit significantly elevated inflammatory responses, including upregulation of IL-6, IL-8, and prostaglandin E2 compared with thermoneutral controls, providing an explanatory cellular mechanism for the clinically observed elevated risks for uterine infections and impaired uterine involution in summer-calving cows [79]. These perturbations produce impaired dominant follicular development accompanied by compromised steroidogenesis in theca and granulosa cells, and elevated levels of FSH through reduced inhibin secretion [73,74,80]. The correlation of increased THI to conception rates has been well established; for every unit increase in THI above 70, the decline in conception rates has been estimated at approximately 4.6% in certain high-producing herds [80], though this relationship varies across regions, breeds, and reproductive management systems [81]. As discussed in Section 2.1, THI does not capture the full spectrum of climatic determinants of fertility; rainfall and wind have been independently documented to influence conception rates in high-producing dairy herds in northeastern Mediterranean environments [82].

#### 2.5.2. Follicular Development and Oocyte Quality

The effects of HS on oocyte quality and competence, as well as embryo dynamics, have been extensively documented across multiple international research groups. The ovarian pool of follicles and their enclosed oocytes is highly sensitive to hyperthermia: heat-induced alterations in small antral follicles can manifest later as compromised maturation and developmental capacity of the ovulating oocyte, with impairment of nuclear and cytoplasmic maturation, mitochondrial dysfunction, induction of apoptotic pathways, and oxidative stress all documented as contributing mechanisms [83,84]. Critically, the effect of HS on oocyte competence is not confined to the summer period; carry-over effects persist into autumn, as evidenced by impaired steroid production, reduced oocyte competence, and lower fertility, with spontaneous recovery requiring two to three estrous cycles after cessation of the thermal insult, indicating that only subpopulations of follicles, rather than the entire ovarian reserve, are damaged upon heat exposure [83]. Mitochondrial dysfunction is now recognized as a central mediator of HS-induced reduction in oocyte developmental competence, with alterations in mitochondrial distribution, membrane potential, ATP production, and mitochondrial DNA copy number all documented under hyperthermic conditions [83]. These findings collectively established the mechanistic framework within which our group has investigated HS effects on oocyte and embryo dynamics under field conditions and in vitro models. Nanas et al. [78], in an in vivo study in Greek Holstein dairy herds, demonstrated that phenotypically thermotolerant and thermosensitive cows exhibit significantly divergent profiles of HSP70, cortisol, progesterone, and conception rate under naturally occurring summer HS conditions, establishing the basis for both herd management and targeted genomic selection. Stamperna et al. [85] demonstrated that during in vitro maturation (IVM), a short-term temperature elevation (41 °C instead of 39 °C from the 2nd until the 8th hour of IVM) significantly reduces embryo yield and induces coordinated gene expression changes, with upregulation of HSP90AA1, G6PD, and CPT1B in cumulus cells, consistent with a metabolic shift toward lipid oxidation for the enclosed oocyte. Blastocysts derived from heat-stressed oocytes exhibit lower DNMT1 and higher PLAC8 expression [86], indicating changes in the epigenomic landscape of the developing embryos; the concurrent upregulation of PLAC8—a gene associated with trophoblast development—may reflect a compensatory response at the embryonic level rather than genuine enhancement of developmental competence, as overall blastocyst yield and quality remain compromised under heat stress conditions. Some of the negative effects of HS were reversed by exogenous HSP70 supplementation to the in vitro culture medium of day-3 embryos from heat-stressed oocytes, significantly improving blastocyst formation rates and total cell numbers, and the results were confirmed across a wider range of thermal challenge protocols [86]. The increased susceptibility of bovine oocytes compared to pre-implantation embryos to heat stress has long been recognized: oocytes at the germinal vesicle and MII stages are substantially more vulnerable than morulae and blastocysts (after the embryonic genome activation), with the developmental stage-specific thermal sensitivity reflecting differences in HSP expression capacity and DNA repair competence [87]. The physiological and genetic determinants of embryonic resistance to elevated temperature, including HSP expression, antioxidant enzyme activity, and membrane lipid composition, represent targets for both selection and intervention to improve embryo survival during summer [88]. Updated reviews of the effects of heat stress on bovine oocytes and early embryonic development confirm that ROS accumulation, spindle disruption, and mitochondrial dysfunction are the central mechanisms of thermal effects at the gamete level [89], with epigenetic changes including altered H3K9 acetylation and global DNA methylation disturbance further compromising the developmental trajectory of the early embryo [90]. The interaction between heat stress and progesterone during early embryonic development is particularly significant: elevated ambient temperature during the first week after fertilization causes luteal insufficiency associated with suboptimal progesterone secretion [78]. This unfavorable endocrine milieu for the early embryo impairs its potential for inteferon-γ synthesis; consequently, the maternal recognition of pregnancy is seriously compromised, and the pregnancy is terminated [91].

#### 2.5.3. Embryo Development and Uterine Environment

Biochemical changes in the follicular fluid of the dominant follicle under HS, including elevated reactive oxygen species, reduced antioxidant enzyme activities, and altered steroid and growth factor profiles, showed that the pre-ovulatory microenvironment is compromised well before thermal insult reaches the oocyte itself [92]. Nanas et al. [93] quantified in vivo that summer HS is associated with significantly more frequent early embryo losses, lower progesterone concentrations, and altered pregnancy-associated glycoprotein (PAG) profiles compared with normothermic controls. Breed-specific responses to heat stress were documented in Holstein versus Limousine in vitro matured oocytes, confirming that HS-induced developmental impairment is not uniform across breeds [86].

#### 2.5.4. Oviductal Alterations and Extracellular Vesicles

The oviductal alterations during summer contributing to the reduced fertility during heat stress periods have been studied using a transcriptomic and structural characterization of the bovine oviducts under HS. RNA sequencing of oviductal epithelial cells from both the ipsilateral and contralateral oviducts to the corpus luteum revealed that HS induces significant transcriptional alterations encompassing EV biogenesis, cytoskeletal organization, and intercellular communication pathways, with concurrent alterations in the concentration of EVs in oviductal luminal fluid [94]. The oviduct communicates with the embryo through its EV cargo, transferring miRNAs, mRNAs, and metabolic enzymes that influence the developmental trajectory of the developing embryo. HS alters the molecular content of the extracellular vesicles (EVs), contributing to the detrimental effects on embryo development caused by direct thermal effects on the embryo itself. This finding provides, to our knowledge, the first molecular evidence in dairy cattle linking HS-induced oviductal transcriptomic alterations to EV-mediated communication with the embryo—an avenue not previously characterized at the transcriptomic and EV level simultaneously. However, the functional consequences of HS-altered oviductal EVs on pre-implantation embryo development remain to be directly demonstrated. Future research should prioritize functional live-cell assays—including co-culture of heat-stressed oviductal epithelial cells with developing embryos and direct application of isolated HS-altered EVs—to establish the mechanistic link between altered EV cargo and impaired embryonic epigenetic programming, implantation success, and offspring phenotype.

#### 2.5.5. Intergenerational Consequences

The intergenerational consequences of gestational HS are significant components of the true cost of HS that is almost under-represented in economic analyses, which to date have focused primarily on direct milk yield losses in the exposed cows [2,3] without accounting for the full multi-generational productive costs. Offspring of heat-stressed dams show reduced birth weight (−4.6 kg), lower weaning weight (−7.1 kg), altered adrenal gland development [95], modified postnatal activity patterns and growth trajectories [96], compromised passive immunity transfer [97], and 8–12% lower milk yield in adulthood. It is likely that these effects are mediated, at least in part, by the increased DNA methylation of the IGF1 gene promoter in mammary tissue, resulting in approximately 20% fewer alveoli at first lactation [98,99,100]. The mechanisms underpinning impaired passive immunity in calves from heat-stressed dams involve both altered colostral immunoglobulin composition and reduced absorptive capacity of the neonatal intestinal epithelium [101], with effects on calf immunity and growth performance confirmed across multiple independent experiments [102]. Heat stress during the dry period affects both dam and calf through hormonal, epigenetic, and immunological pathways operating in parallel, with carry-over effects on the magnitude and pattern of the subsequent lactation curve [103,104]. In utero thermal exposure further alters the liver transcriptome and DNA methylation landscape of calves, with enrichment in pathways governing lipid metabolism, gluconeogenesis, and inflammatory signaling that may predispose to metabolic dysfunction in adult life [105]. Exposure of dry cows to elevated temperatures have significant carry-over effects even to the second generation: granddaughters of heat-stressed cows produce approximately 2.8 lb/day less milk in their first lactation [99,100], suggesting that, in chronically heat-stressed populations, the cumulative productive cost of repeated summer heat-stress events may continue to be expressed through the performance of daughters and granddaughters over a period of 3.5–4 years, as documented in studies under sustained thermal challenge [96,97]. Halli et al. [106] demonstrated that HS-induced methylation, transcriptomic, and metabolomic signatures are reliably transmitted to F1 calves. Early-life heat stress during the neonatal period compounds these in utero programming effects by impairing thermoregulatory capacity and feeding behavior [40]. Cooling interventions must therefore extend to dry and transition cows: heat stress during the dry period directly suppresses mammary gland development [107], while comprehensive pre- and postnatal cooling significantly improves offspring thermoregulation, immunoglobulin absorption, and growth performance [108]. From a management perspective, the evidence collectively supports that cooling interventions must extend to dry and transition cows to capture the full intergenerational productive benefit [10]. Reproductive management strategies that synchronize ovulation, supplement progesterone during early gestation, and embryo transfer have demonstrated efficacy in recovering conception rates under summer conditions [109,110]. Management of heat stress at the herd level in subtropical environments has confirmed that cooling programs can improve conception rates, but it is unlikely to reach the winter levels [111]. These findings are indicative of the epigenetic carry-over effects of heat stress, which worsen in chronically heat-stressed dairy populations.

## 3. Microbiome Dynamics Under Heat Stress: From Rumen Dysbiosis to Systemic Endotoxaemia

### 3.1. The Rumen Microbiome as Active Pathophysiological Amplifier

The rumen harbors an estimated 10^10^–10^11^ microbial cells/mL of fluid, which are responsible for lignocellulose fermentation, SCFA production, and microbial protein synthesis that accounts for approximately 70% of the cow’s metabolism [100,112]. Traditionally, the rumen microbiome is affected, rather than contributing, to HS pathology. A growing body of evidence argues that rumen microbiome shifts are associated with amplification of the productive and health consequences of HS, though causal directionality remains to be fully established [113,114]. Primarily, the loss of butyrate-producing taxa reduces luminal butyrate, depleting the primary nutrients of colonocytes, compromising barrier maintenance function, and influencing anti-inflammatory signals that normally restrain NF-κB activity in intestinal immune cells [34]. A systematic review of heat stress effects on rumen epithelium and fermentation confirmed that papillae morphology, tight junction protein expression, and volatile fatty acid absorption capacity are all significantly impaired under thermal challenge, with a positive correlation between severity of fermentative disruption and HS intensity and duration [115]. The characterization of rumen microbiome and fatty acid profiles under heat stress confirmed reductions in total SCFA concentration, acetate:propionate ratio, and butyrate production capacity. On the other hand, an increase in lactate and valerate is indicative of a shift toward more acidogenic fermentation pathways, which appear to be less efficient [116]. Second, the enrichment of lactate-producing Streptococcus and amylolytic taxa leads to pH reduction in the rumen [117,118]. Third, structural disruption of intestinal epithelial tight junctions under HS-induced splanchnic ischemia enables translocation of rumen LPS and microbial fragments into systemic circulation, initiating the chronic endotoxemia that amplifies the HS-driven immune dysfunction [19,20,21].

### 3.2. Compositional Shifts During HS

Metagenomics and 16S rRNA gene sequencing studies have established a HS-associated compositional signature in the bovine rumen microbiome; yet there are many factors, such as the HS intensity and duration, breed, diet and physiological stage, that affect the magnitude and specific taxa [119,120]. The most consistent changes concern the suppression of *Butyrivibrio*, *Pseudobutyrivibrio*, *Selenomonas*, *Prevotella*, and *Acetobacter*, the enrichment of lactate-producing Streptococcus and amylolytic Ruminobacter and Treponema and the increased presence of pathogenic taxa [113,114]. Feng et al. [120] reported that rumen community structure was most severely disrupted in growing heifers and least affected in lactating cows, exhibiting a stage-specific vulnerability. Li et al. [114] demonstrated that heat-resistant animals are characterized by different compositions in their microbiome, dominated by *Ruminococcus flavefaciens* and *Succiniclasticum*, arguing that rumen microbiome composition may not be solely a consequence of HS, but could act as a contributing factor to phenotypic thermotolerance—an association that warrants functional validation. Koch et al. [121] characterized the combined effects of HS and altered feeding patterns on the microbiome of the jejunum alongside plasma and fluid metabolites, revealing that HS independently disrupts intestinal microbial community structure and metabolite profiles in ways that cannot be attributed to reduced feed intake alone. Breed-specific rumen microbiome shifts under heat stress have been confirmed across Holstein and mixed-breed populations, with differential abundances in *Prevotella*, *Butyrivibrio*, and *Lachnospiraceae* members correlating significantly with breed-level thermotolerance phenotypes [122].

### 3.3. The Milk Microbiome as a Thermal Status Biomarker

Under thermoneutral conditions, the bovine milk microbiome is dominated by commensal genera including *Staphylococcus*, *Streptococcus*, *Lactobacillus*, and *Corynebacterium*, which maintain a stable microbial environment, which indicates mammary gland’s health [123,124]. Heat stress disrupts this equilibrium through multiple mechanisms: reduced blood flow to the mammary gland compromises its immunity by affecting the delivery of immunoglobulins, lactoferrin, and immune cells to the gland, creating a permissive environment for microbial expansion, while the suppression of neutrophil and macrophage function reduces bacterial clearance at the teat canal [38,71]. The effects of heat stress in the bovine milk microbiome of Holstein and Brown Swiss were examined, and it was shown that HS significantly altered milk microbiota composition with greater disruption in Holstein cows (74 Operational Taxonomic Units (OTUs) changed) than Brown Swiss (20 OTUs), consistent with the established higher thermotolerance of the Brown Swiss breed. The most affected genera in Holstein cows included *Acinetobacter*, *Chryseobacterium*, *Enterococcus*, *Lactococcus*, *Serratia*, and *Streptococcus*. These shifts were also maintained during the autumn recovery periods, suggesting that HS is responsible for microbial imprints, analogous to the epigenetic carry-over effects [125]. The relationship between milk microbiome dysbiosis and subclinical mastitis under HS warrants particular attention. While overt clinical mastitis episodes increase 2–3 fold during summer, subclinical intramammary infections—detectable through elevated somatic cell counts (SCC) and altered microbiome composition, but without clinical signs—represent a substantially larger and often underappreciated burden on milk quality and cow welfare [52]. Microbiome-targeted interventions, including teat dipping protocols optimized for summer pathogen profiles, probiotic supplementation aimed at restoring commensal dominance, and nutritional strategies supporting mammary immune defense, represent promising solutions for mitigating the milk quality consequences of HS-induced mammary dysbiosis [34,126].

### 3.4. The Leaky Gut Axis: Intestinal Barrier Failure and Systemic Inflammation

Under HS, the shift in blood circulation towards skin vessels reduces splanchnic perfusion and creates intestinal ischemia that compromises tight junction proteins, such as claudins, occludin, and ZO-1, enabling LPS translocation [19,127,128]. Pearce et al. [21] provided the first evidence that HS causes infiltration of the small intestinal jejunum by a previously uncharacterized macrophage-like immune cell population, with transcriptomic disruption of epithelial cell identity genes attributable directly to hyperthermia rather than to reduced DMI. Fontoura et al. [20], by evaluating chromium EDTA plasma concentrations, quantified the increase in total-tract gut permeability in heat-stressed Holstein cows and demonstrated that dietary organic acid and pure botanical (OA/PB) supplementation consisting of citric acid, sorbic acid, thymol, vanillin and triglycerides partially restores both gut barrier function and lactation performance. Bacillus subtilis probiotic supplementation, which reduces plasma LPS by 50% and IL-6 by 20% in heat-stressed dairy cows [34], proves that this microbiome–leaky gut–inflammation axis is a target of management towards thermoresilience. Furthermore, microbial extracellular vesicles are increasingly recognized as mediators of microbiome–host communication through cargo transfer of miRNAs, enzymes, and bioactive lipids [129].

## 4. Multi-Omics Insights into the Molecular Architecture of Heat Stress

### 4.1. The Systems Biology Imperative: Why Single-Omics Is Insufficient

It is now clear that the biological response of dairy cattle to HS is genuinely systemic, involving coordinated changes in gene transcription and translation and epigenetic regulation across multiple tissues, cell types, and time scales [34,130]. An integrative metabolic omics study of heat-stressed cows revealed reduced lipolysis, increased glycolysis, and amino acid catabolism at the organismal level, with impaired mammary epithelial cell function and mTOR signaling at the cellular level [131]. The subsequent application of gene–metabolite correlation analysis revealed regulatory axes enriched in sphingolipid signaling and arachidonic acid metabolism that are not detectable in transcriptomics or metabolomics separately [114]. This means that our understanding of HS molecular mechanisms is incomplete when individual omics platforms are analyzed separately, and that integrated multi-omics approaches represent a powerful analytical framework for evaluating the effects of HS at the molecular level.

The molecular response to HS operates as a hierarchical cascade across omics layers. Genomic variation in thermotolerance loci determines individual adaptive capacity and sets the ceiling of the physiological response. This genomic architecture is reflected in coordinated transcriptomic reprogramming across multiple tissues, encompassing upregulation of stress-response networks alongside suppression of productive and immune gene programs. These transcriptional changes are selectively translated into proteomic reconfigurations that modify cellular function independently of mRNA abundance. The metabolome represents the integrated biochemical output of all upstream layers, capturing the net consequence of genomic, transcriptomic, and proteomic dysregulation on energy metabolism, antioxidant capacity, and immune function. No single omics layer captures the full regulatory architecture of this response; their integration is therefore a biological necessity rather than a methodological preference. However, the implementation of integrated multi-omics approaches remains challenging. Simultaneous generation of genomic, transcriptomic, proteomic, and metabolomic datasets is costly and limits large-scale studies. Additional barriers include the lack of standardized field sampling protocols, batch effects across platforms, and the computational complexity of data integration. Publicly available multi-omics datasets specific to heat-stressed dairy cattle remain scarce, hampering meta-analytic approaches. Coordinated international efforts and shared data infrastructures will be essential to overcome these limitations.

### 4.2. Genomics: The Genetic Architecture of Thermotolerance

Heat tolerance in dairy cattle is a complex trait with low heritability (h^2^ ≈ 0.10–0.20 for milk yield-based slope traits in reaction norm models), with heritabilities of genetic components varying with THI level from 0.17 to 0.47 for milk yield and from 0.08 to 0.55 for conception rate as THI increases [10,132,133]. The negative genetic correlation between milk yield potential and the THI threshold for productive decline (r ≈ −0.53) reflects the fundamental physiological antagonism between high productive yields and efficient thermoregulation [10,11], implying that selection indices must consider this genetic correlation for the successful application of breeding programs by balancing genetic gains and resilience to heat stress.

Genome-wide analysis of 40,000 animals identified candidate causal variants in neuronal signaling genes, most notably *ITPR1*, *ITPR2*, *NPFFR2*, and *CALCR* [134], whose inclusion in the 50 k SNP panel improved heat tolerance prediction accuracy by up to 10% [10]. Development of genomic predictions for heat tolerance using ssGBLUP across more than 2 million genotyped US Holstein animals has confirmed the feasibility of national-scale genomic evaluation for heat tolerance traits [133]. A comprehensive candidate gene analysis identified the HSP gene family—*HSPA1A*, *HSPA1B*, *HSPA1L*, *HSP90AB1*, *GRXCR1*, and *FKBP4*—and DNAJ chaperone members through selection signature analyses in tropically adapted Bos indicus populations [72] while identifying *HSF1* as a causal variant and *MGST1* as a candidate associated with rectal temperature and respiration rate in US Holsteins [135,136]. The extent of the transcriptomic and genomic response to HS, involving hundreds of differentially expressed genes across immune, metabolic, and structural families, proves that thermotolerance is not a single-gene trait, but a property of the entire genome [72]. CRISPR-Cas9 technologies for livestock precision breeding have advanced rapidly and now offer plausible routes to introducing thermotolerance alleles with minimal off-target effects and without the generation interval constraints of conventional crossbreeding [137]. Introduction of the Senepol slick-hair allele at the prolactin receptor (*PRLR*) locus into Holstein backgrounds utilizing conventional reproductive methods demonstrated improved thermoregulatory capacity without the milk yield dilution associated with conventional crossbreeding [138]. The same results were observed in Angus and Jersey breeds, where gene editing technologies (CRISPR/Cas9) were used to introduce these PRLR alleles, improving the regulation of vaginal and rectal temperatures of heifers and bulls, respectively, while also positively affecting growth characteristics [139]. These gene editing technologies received the first FDA low-risk determination for a food animal intentional genomic alteration in 2022 [140]. Strategies for countering heat stress through selective breeding for temperature regulation, exploitation of the biology of heat tolerance, and molecular biology tools were conceptually outlined in early genomics-era work that anticipated many of the candidate gene discoveries since validated by GWAS [141]. Whole-genome sequencing of phenotypically thermotolerant and thermosensitive Holstein cows identified genome-wide genetic variation and revealed pathways governing the Heat Shock Response, Unfolded Protein Response, and systemic immune activation as central to thermotolerance, with SNPs in five HSP-encoding genes emerging as candidate markers for pre-selection characterization of calves and embryos [142].

### 4.3. Transcriptomics: Multi-Tissue Resolution of the HS Gene Expression Landscape

RNA-seq has been used to detect gene expression changes under HS across multiple tissues including PBMCs, mammary epithelial cells, liver, hypothalamus, rumen epithelium, and oviductal epithelial cells [34,94,114,118,143,144]. In PBMCs, HS induces upregulation of *IL6, TNF, IL1B*, and *NOX*, alongside downregulation of *SOD1* and *GPX1* [34,51]. In mammary cells, casein gene family suppression accompanies upregulation of *HSPA1A*, *HSPA1B*, and *HSP90AA1* [34,144]. Liver transcriptomics under HS revealed upregulation of acute-phase response and gluconeogenesis pathways alongside suppression of lipogenic networks [143]. In utero thermal exposure leaves a lasting transcriptomic imprint in the liver of dairy calves, with differentially expressed genes enriched in fatty acid oxidation, gluconeogenesis, and inflammatory pathways that may program metabolic phenotype from birth [105]. Integration of host transcriptomics with rumen metagenomics identified *RAB39B*, a vesicular trafficking gene, as significantly associated with rectal temperature and respiration rate [145]. The ceRNA network profiling of the hypothalamic-pituitary-mammary gland axis identified coordinated regulatory circuits under HS [146]. Transcriptomic characterization of the bovine oviduct under HS revealed transcriptional alterations encompassing EV biogenesis, cytoskeletal organization, and molecular communication pathways, with concurrent changes in oviductal luminal EV concentrations [91]. Mild heat stress triggers an adaptive transcriptomic response in blood mononuclear cells and mesenteric lymph node leukocytes characterized by upregulation of HSP chaperones, antioxidant enzymes, and anti-apoptotic signals—a response that differs qualitatively from the maladaptive immune suppression documented under severe HS [57]. Identification of heat stress-responsive microRNA miR-541 in bovine mammary epithelial cells, and its role in post-transcriptional regulation of *HSP27* expression, illustrates that non-coding RNAs add a further dimension of complexity to the transcriptomic HS response that cannot be captured by mRNA profiling alone [147]. The first snRNA-seq characterization of the heat-stressed bovine mammary gland [148] revealed cell-type-specific transcriptomic responses inaccessible to bulk RNA-seq.

### 4.4. Proteomics: Capturing Post-Translational Regulation

Proteomics confirmed elevated acute-phase proteins (haptoglobin, SAA, and fibrinogen) and reduced antioxidant defense proteins (SOD, GPx, and thioredoxin) in heat-stressed dairy cattle [34,54]. Skibiel et al. [149] provided a deep characterization of dry-period HS carry-over effects: phosphoproteomics of the lactating mammary gland at 14, 42, and 84 DIM identified 251 proteins with differential abundance and 224 proteins with differential phosphorylated status, with pathway enrichment in epithelial adherents junction remodeling, RhoGDI signaling, microtubule dynamics, and cytoskeletal organization. Zachut et al. [50] identified 107 differentially abundant proteins enriched toward Nrf2-mediated oxidative stress response pathways in adipose tissue of late-pregnant HS cows. An integrated plasma metabolomics and proteomics approach applied to Holstein dairy cows with different resilience to HS identified specific biomarkers, including lipid metabolism intermediates, amino acid derivatives, and stress-response proteins. These markers could be further utilized for thermal status classification, supporting the use of multi-platform omics profiling as a precision tool for herd-level thermotolerance monitoring [114]. An integrated plasma proteomics-metabolomics comparison of heat-tolerant and heat-susceptible Holstein cows [114] identified 3-methoxytyramine and (3Z)-phytochromobilin as plasma biomarkers achieving AUC values exceeding 0.88 for thermotolerance phenotype discrimination.

### 4.5. Metabolomics: The Biochemical Fingerprint of a Compromised Animal

Liquid or gas chromatography coupled to mass spectrometry (eg GC-MS and LC-MS/MS) metabolomic studies consistently document elevated β-hydroxybutyrate (BHB), lactate, and pyruvate reflecting energy deficiency and anaerobic glycolysis, reduced NEFA and glucose confirming the paradoxical metabolic phenotype mentioned above; depleted glutathione and ascorbate indicating reduced antioxidant capacity, elevated cortisol confirming HPA axis activation; and altered free amino acid profiles consistent with catabolism for gluconeogenesis and HSP synthesis at the expense of mammary protein production [34,150]. Blood amino acid profiling under HS reveals consistent depression of branched-chain amino acids (leucine, isoleucine, and valine) alongside increases in aromatic amino acids and sulphur-containing intermediates, a pattern that reflects both the diversion of AAs toward HSP synthesis and the suppression of mammary mTOR-mediated protein anabolism [150]. Meta-analysis of the effects of heat stress on feed intake, milk yield, milk composition, and feed efficiency across 50 studies confirms that milk protein and fat percentages are significantly depressed alongside yield, and that efficiency losses are proportionally greater than yield losses—indicating that the metabolic cost of HS falls disproportionately on nutrient conversion rather than simply on intake [12]. Jorge-Smeding et al. [151] characterized plasma and milk metabolomics changes in Holstein cows under HS, revealing significant alterations in amino acid metabolism pathways that directly connect systemic metabolic disruption to milk protein composition changes. Kim et al. [55] studied transcriptomic, plasma metabolomic, and gut microbiome alterations during HS, identifying nine differential plasma metabolites alongside 226 DEGs and 18 significantly altered gut microbiome genera. The rumen metabolome is equally disrupted: LC-MS profiling identified 1065 and 571 differential metabolites in heat-stressed Holstein and Jersey cows, respectively [114].

### 4.6. Epigenomics: Scale, Transgenerational Transmissibility, and Nutritional Modifiability

The epigenomic dimension of HS represents one of the most significant recent developments in the field, because the epigenetic reprogramming under HS, and its potential for transgenerational inheritance, fundamentally reframes the question of what the real HS cost is and whether cooling interventions prevent or merely delay its consequences. A genome-wide bisulfite sequencing study [152] identified 49,861 DMRs corresponding to 7613 differentially methylated genes between spring and summer blood samples from dairy cows, with promoter-associated DMGs enriched in ROS metabolism, signal transduction, and energy metabolism. Multi-generational multi-omics analysis demonstrated that HS induces transmissible methylation, transcriptomic, and metabolomic patterns in Holstein dams and their female calves [106]. An independent study [153] identified HS-specific DMPs in the *BCL2L12* anti-apoptotic promoter in high immune responders. 5-azacitidine demethylation experiments in Bos indicus vs. crossbred cattle [154] confirmed that promoter hypomethylation is essential for *HSP70*, *HSP90*, and *STIP1* expression. The magnitude of these epigenomic consequences must be appreciated against the fact of a warming climate in which HS events are increasing in frequency, intensity, and duration [7]: projected increases in the number of days per year at which dairy cattle experience heat stress hazard conditions [4] suggest that the cumulative epigenetic burden on cattle populations may increase as thermal events become more frequent, though the transgenerational compounding of these effects remains to be empirically quantified. The nutritional interventions used to tackle the undesired epigenetic changes on DNA methylation patterns οpens a potentially promising strategy for epigenetic mitigation in the HS context, though direct evidence in heat-stressed dairy cattle remains limited [155].

Concerning the non-coding RNAs, miRNA-541 modulates *HSP27* expression through ceRNA interactions [156]; N6-methyladenosine (m6A) RNA modification is reprogrammed transcriptome-wide in bovine mammary epithelial cells under HS, with functional consequences for the stability and translation efficiency of stress-responsive transcripts [157]; and abnormal *IGF1* gene promoter methylation in mammary tissue of offspring of heat-stressed dams—reducing alveolar development by approximately 20% and milk yield by 8–12% in adulthood—represents the most clinically significant epigenetic consequence of gestational HS yet identified [98,99,158].

### 4.7. Integrative Multi-Omics: Toward a Systems Model of Thermal Stress

Li et al. [114] uncovered 411 significant gene–metabolite correlations enriched in sphingolipid signaling and arachidonic acid metabolism through integrated analysis. Du et al. [159] demonstrated that Saccharomyces cerevisiae culture supplementation mitigates HS-related damage through multi-omics-characterized changes spanning rumen microbiome stabilization, metabolite profile improvement, and blood parameter amelioration—an example of multi-omics integration towards nutritional intervention efficacy. The integration of multi-omics data with real-time IoT sensor streams and AI-driven predictive models represents the frontier of precision HS management [22,34,160]. Single-cell RNA sequencing and spatial transcriptomics for bovine mammary and rumen tissues are methodological frontiers whose application to bovine HS biology has barely begun.

## 5. Mitigation Strategies: Evidence, Mechanisms, and Implementation

### 5.1. Environmental and Management Interventions

Evaporative cooling systems combining ventilation fans with water sprinklers or misters remain the most immediately effective approach to reducing THI in intensive housing systems [22,161]. Mechanistic thermoregulatory modeling confirmed that fan cooling alone is effective at ambient temperatures below 26 °C, but requires the combination of fans and sprinklers for effective HS alleviation at higher temperatures [162]. Tunnel-ventilated barns with evaporative cooling pads reduce THI by up to 4.9 units, decreasing rectal temperatures by 0.4–1.0 °C and increasing milk yield by 0.3–5.3 kg/day [163], though retrofitted tunnel barns perform substantially less well than purpose-built ones [164]. Shade provision independently reduces thermal load in cows managed on pasture or in open-lot systems: the quantity and quality of shade significantly influence both physiological indicators of heat stress and time spent at the feed bunk, lying, and ruminating, with cows deprived of shade showing higher respiration rates, greater cortisol concentrations, and reduced DMI [165]. Practical frameworks for detecting and abating heat load at the herd level—combining behavioral monitoring, sensor-based heat load indices, and decision-support tools—have been developed for implementation across diverse production systems [166]. For dairy producers in the humid tropics and developing-world countries, where access to mechanical cooling infrastructure is limited, management of feeding time, housing orientation, and access to natural shade remains the primary heat mitigation toolkit [167]; in temperate dairy industries facing climate change, the challenge is reconfiguring existing facilities and management systems to cope with thermal events that exceed their original design parameters [8].

A precision cooling approach centered on behaviorally informed minimal water delivery has demonstrated preliminary efficacy in maintaining animal comfort indicators while substantially reducing water and energy consumption [158]—addressing growing sustainability concerns about the water footprint of evaporative cooling. The application of machine learning early warning systems trained on sensors incorporating THI, respiration rate, body temperature, lying time, and activity enables automated cooling system activation before productive losses materialize [22,160]. Integrated monitoring of herd-level welfare indicators within a THI-productivity surveillance framework enables early herd-level HS detection [168].

### 5.2. Nutritional Interventions: Targeting the Specific Metabolic Deficits of the Heat-Stressed Cow

Before reviewing specific interventions, it should be noted that most nutritional studies in this section were not conducted under pair-feeding conditions. Consequently, it remains unclear in many cases whether observed improvements in performance reflect direct mitigation of cellular heat stress or are partly attributable to increased feed intake. Future studies incorporating PFTN designs are needed to disentangle these effects and provide robust nutritional recommendations.

#### 5.2.1. Amino Acids, Electrolytes, and Methyl Donors

Nutritional strategies for managing cattle under thermal stress were first systematically proposed by Beede and Collier [169], whose fundamental work on electrolyte supplementation and dietary energy density under heat stress continues to facilitate modern feeding programs. The understanding of HS-induced mTOR suppression, AA catabolism, and mammary AA transporter downregulation provides a basis for targeted AA supplementation. The supplementation of protected methionine showed promising results, since it improves milk protein synthesis by 12–15% in heat-stressed cows, affecting substrate availability, antioxidant precursor supply, and enhancing mTOR pathway [34,170]. Dietary Cation–Anion Difference (DCAD) manipulation in feed stabilizes blood pH within 7.35–7.45 while significantly increasing DMI by 8–10% [34,171]. Saturated fatty acid supplementation in heat-stressed dairy cows in mid-lactation partially offsets the reduced energy intake by providing a dense energy source, improving milk fat content and energy balance without the thermogenic characteristics of fermentable carbohydrate [172]. Betaine reduces the energetic cost of cellular volume maintenance under thermal stress, serves as a chaperone assisting in the refolding of heat-denatured proteins, and redirects methionine towards catabolic use [173,174,175]. A controlled evaluation of betaine effects in lactating Holstein cows subjected to heat stress confirmed that betaine improved DMI, energy corrected milk yield (ECM), and nitrogen efficiency, supporting its inclusion in summer feeding programs for high-producing cows [176]. Field studies in grazing dairy cows demonstrated betaine supplementation increased milk yield by over 6% during periods of high nocturnal THI [173], while controlled climate chamber experiments found betaine reduced maximum body temperature during thermoneutral conditions but did not provide direct thermal mitigation at peak heat challenge, indicating its protective role is exhibited mainly during the recovery phases rather than in the acute phases of HS [174]. Betaine effects on body temperature indices, performance, and hematological variables have also been evaluated in dairy heifer calves under hot summer conditions, with significant improvements in feed efficiency and reductions in rectal temperature and respiration rate supporting its use across age groups [177]. The dose-dependence of betaine effects and its interaction with fat supplementation (which can increase body temperature through the heat of lipid oxidation) require careful consideration in practical feeding program design [174]. Comprehensive nutritional intervention reviews confirm that no single strategy fully compensates for HS-induced productive losses; rather, combined approaches targeting DMI maintenance, metabolic pH buffering, antioxidant status, and mTOR signaling are needed to address the multi-mechanistic nature of HS pathophysiology [47].

#### 5.2.2. Antioxidants and Microminerals

The oxidative stress documented under HS provides a rationale for antioxidant supplementation, though the results vary considerably across studies [34]. Omega-3 polyunsaturated fatty acid supplementation impacts the immune system’s functionality by reducing circulating IL-6 and TNF-α by approximately 40% through NF-κB competitive inhibition and has demonstrated reductions in inflammatory markers in heat-stressed calves [34,178]. Astragalus polysaccharide supplementation activates the Nrf2/ARE pathway, increasing SOD and catalase activities by 15–20% [34]. Phytogenic extracts including capsaicin have demonstrated reductions in surface temperature and improvements in time at the feeding line [158]. Among microminerals, organic selenium enhances GPx activity and has maintained antioxidant capacity in heat-stressed Holstein cows where inorganic selenium groups showed progressive decline [179,180]. The broad evidence base for micromineral supplementation under heat stress—spanning selenium, zinc, copper, manganese, and chromium—has been reviewed in the context of their roles in antioxidant enzyme cofactor supply, immune modulation, and reproductive function, with practical recommendations for organic versus inorganic sources differing significantly in bioavailability under the altered rumen fermentation conditions of HS [181]. The interaction between nutritional status, immune function, and health in dairy cattle is complex and context-dependent: high-yielding cows experience a convergence of metabolic stress, immune challenge, and thermal load in summer that requires nutritional strategies addressing all three axes simultaneously [182]. Comprehensive nutritional strategy reviews for dairy ruminants under heat stress conditions have identified synergistic approaches combining antioxidants, osmolytes, and immune modulators as superior to single-supplement protocols [183]. Chromium yeast improved welfare indicators, increased DMI, and improved antioxidant and immune function in mid-lactating dairy cows under HS [183]. Selenium and vitamin E combined supplementation improved immune function, reproductive performance, and milk yield in transition dairy cows in subtropical conditions [184], while combined trace mineral supplementation (Se, Zn, and Cu) reduced HS-associated oxidative stress and metabolic alteration [185]. Evidence quality across these micromineral studies varies considerably, with many lacking PFTN controls.

#### 5.2.3. Microbiome-Targeted Nutritional Strategies

Bacillus subtilis probiotic supplementation reduces plasma LPS by 50% and IL-6 by 20% in heat-stressed dairy cows [34]—the strongest direct evidence that the gut–mammary endotoxemia axis is therapeutically tractable. Saccharomyces cerevisiae culture stabilizes rumen pH and has been characterized at the multi-omics level by Du et al. [156] as mitigating HS-related damage through coordinated rumen microbiome stabilization, metabolite profile improvement, and blood parameter amelioration. Earlier controlled trials with supplemental yeast culture in heat-stressed lactating Holstein cows demonstrated improvements in milk yield and reductions in body temperature during peak afternoon heat, consistent with a rumen pH-stabilizing mechanism [186]. OA/PB supplementation partially restores intestinal barrier integrity and lactation performance during HS [20]. Multi-omics-guided precision nutrition represents the frontier of evidence-based nutritional HS management, currently constrained by practical barriers in high-throughput metabolomics but increasingly feasible as platform costs decline [34,151].

### 5.3. Genetic Improvement and Genomic Selection: Long-Term Solutions

Genetic and genomic improvement represent the only strategies with the potential to sustainably reduce the intrinsic physiological vulnerability of dairy cattle to thermal challenge. Incorporating functionally prioritized SNPs from ITPR1, ITPR2, NPFFR2, and CALCR into the 50 k SNP panel improved heat tolerance prediction accuracy by up to 10 percentage units [10]; ssGBLUP applications across >2 million genotyped animals have confirmed the feasibility of national-scale genomic evaluation [133]. ICAR has incorporated these molecular markers into the global genetic evaluation system, enabling cross-border diffusion of heat-tolerant genetics through international frozen semen trade [11,34]. The urgency of this genetic work is underscored by projected increases in heat stress hazard exposure for European cattle under all emissions scenarios: mapping of heat stress hazard under current and future climate conditions identifies large areas of currently temperate dairy production where thermal challenge will reach current subtropical levels within decades [4], with consequences for animal welfare and productive efficiency that justify long-term investment in genomic thermotolerance. At the breed level, systematic crossbreeding with heat-adapted tropical breeds—Senepol (*PRLR* slick-hair), Brahman (*MC1R*), and Zebu breeds (elevated *HSPB1*, *DNAJC18*, and *HSPA4*)—produces F1 offspring with substantially improved thermoregulatory indices while maintaining commercially competitive milk yields [34,135,136]. Introduction of the slick-hair allele [138] and multi-locus editing of *HSPA1A* + *SOD2* [34] represent gene-editing routes to thermotolerance improvement pending regulatory framework development. The most durable long-term improvement will emerge from multi-trait genomic selection indices explicitly balancing heat tolerance against milk production, fertility, health, and longevity [10,132].

The implementation of these mitigation strategies varies considerably across production systems. Evaporative cooling infrastructure, precision monitoring technologies, and genomic selection programs are primarily accessible to large-scale intensive operations in high-income countries, where capital investment and technical expertise are available. Smallholder and extensive systems—which account for most of the dairy production in tropical and subtropical regions most severely affected by HS—face significant barriers to adoption, including high upfront costs, limited veterinary and nutritional advisory services, and lack of access to genomic tools. Nutritional interventions such as betaine, organic selenium, and yeast culture represent more scalable options for resource-limited settings, though their cost-effectiveness under field conditions without PFTN validation remains uncertain. Bridging this implementation gap will require region-specific adaptation of mitigation frameworks, targeted subsidies, and capacity-building initiatives aligned with national agricultural policy.

## 6. Conclusions

Table 1 summarizes the major biological systems affected by HS in dairy cows and their interconnections with production outcomes.

This review has provided strong evidence that the biological consequences of heat stress in dairy cows are substantially more complex, more epigenetically consequential, and more amenable to mechanistically targeted intervention than conventional single-domain analyses have allowed. Our conclusions are summarized on four major scales.

First, HS is not a feeding problem and treating it as one has been a strategic error that has misdirected both research and management resources for decades. The PFTN paradigm established irrefutably that 50–65% of productive losses arise from direct hyperthermia-driven effects on tissue physiology—mTOR suppression, the insulin–glucose paradox, and somatotropic axis uncoupling—that are completely independent of DMI and cannot be recovered by any intervention that targets DMI alone. Nutritional strategies designed for thermoneutral NEB are mechanistically inappropriate for HS NEB, and their continued application without modification perpetuates preventable productive losses.

Second, the rumen and gut microbiome are active, not passive, participants in HS pathophysiology. HS-induced dysbiosis—loss of butyrate-producing taxa, enrichment of lactate producers, compromised rumen epithelial barrier, intestinal tight junction failure, and the resulting endotoxaemia—is associated with a self-reinforcing inflammatory cycle that may chronically amplify other dimensions of HS damage. The discovery that rumen microbiome composition partially mediates individual differences in thermal tolerance, and that milk microbiome profiling can distinguish thermal comfort from HS conditions with random-forest accuracy, opens genuinely new avenues for precision monitoring and microbiome-targeted therapy.

Third, the epigenomic dimension of HS demands a fundamental recalibration of how we measure HS effects in the long term. Nearly 50,000 differentially methylated regions across the genome under summer HS; multi-omics signatures transmitted with demonstrable fidelity to F1 calves; these findings mean that the economic cost of a heat-stress event continues to be expressed through the productive performance of daughters and granddaughters for 3.5–4 years after the event itself. Conventional economic analyses that measure only the milk yield loss in the directly exposed cow systematically underestimate the true cost by an unknown but potentially very large factor.

Fourth, the oviductal transcriptome and EV communication axis identified by Stamperna et al. [91] provides, for the first time, a plausible molecular mechanism for the persistence of summer subfertility well beyond the thermal insult. This finding reframes the oviduct from a passive reproductive conduit to an active HS-sensitive signaling organ whose disrupted EV output may compromise embryonic epigenetic programming and developmental trajectory. The functional consequences of HS-altered oviductal EVs for pre-implantation embryo development, implantation success, and offspring phenotype represent one of the highest-priority research questions in this field.

### Future Directions and Research Priorities

Looking forward, we identify five priority areas for the field. First, multi-tissue, multi-breed, longitudinal studies combining rumen metagenomics with host transcriptomics, proteomics, and metabolomics are needed to establish causal microbiome–host interactions rather than associations. Second, functional live-cell assays characterizing the effect of HS-altered oviductal EVs on pre-implantation embryo development and epigenetic programming should be prioritized, moving beyond descriptive transcriptomics. Third, AI-driven integration of multi-omics datasets with real-time IoT sensor streams should be developed into validated decision-support tools deployable at the herd level. Fourth, regulatory frameworks for responsible clinical deployment of CRISPR-based thermotolerance improvements need to be established in parallel with the science, not after it. Fifth, nutritional epigenetics interventions—particularly methyl donors and micronutrients targeting HS-induced DNA methylation changes—represent a largely unexplored avenue with immediate practical potential. At the policy level, equitable access to cooling infrastructure, genomic tools, and evidence-based nutritional strategies across smallholder and intensive production systems globally must become an explicit goal of national and international agricultural policy.

## Figures and Tables

**Table 1:** Overview of the major biological systems affected by heat stress in dairy cows, their key molecular and physiological alterations, downstream consequences, and associated production impacts.

System	Key HS-Induced Changes	Downstream Consequences	Production Impact
**Neuroendocrine**	GnRH suppression, cortisol hypersecretion, and reduced LH surge	Anovulation, delayed ovulation, and corpus luteum regression	Reduced conception rates and impaired fertility
**Metabolic**	Reduced DMI, mTOR suppression, increased glycolysis, and amino acid catabolism	Negative energy balance and reduced milk protein synthesis	Milk yield decline (50–65% direct thermal effect)
**Gut barrier and microbiome**	Tight junction disruption, LPS translocation, and rumen dysbiosis	Systemic endotoxemia, chronic inflammation, and altered VFA production	Reduced DMI and impaired immune function
**Immune**	NF-κB activation, elevated IL-6/TNF-α, and neutrophil dysfunction	Increased mastitis/metritis risk and impaired passive immunity transfer	Health costs and reproductive losses
**Reproductive**	Follicular dysfunction, oocyte mitochondrial damage, and oviductal EV alterations	Reduced oocyte competence, embryo losses, and impaired maternal recognition	Lower pregnancy rates and intergenerational effects
**Epigenomic**	DNA methylation changes, altered miRNA profiles, and m6A reprogramming	Transgenerational transmission of HS signatures and *IGF1* promoter methylation	8–12% lower milk yield in offspring

## Data Availability

No new data were created or analyzed in this study. Data sharing is not applicable to this article.

## Abbreviations

The following abbreviations are used in this manuscript:

AMPK	AMP-Activated Protein Kinase
BCS	Body Condition Score
BHBA	β-Hydroxybutyrate
DAMP	Danger-Associated Molecular Pattern
DMI	Dry Matter Intake
DMRs	Differentially Methylated Regions
EVs	Extracellular Vesicles
FSH	Follicle-Stimulating Hormone
GH	Growth Hormone
GnRH	Gonadotrophin-Releasing Hormone
HPA	Hypothalamus–Pituitary–Adrenal
HS	Heat Stress
LH	Luteinizing Hormone
MAPK	Mitogen-Activated Protein Kinase
mTOR	Mechanistic Target of Rapamycin
NEB	Negative Energy Balance
NEFA	Non-Esterified Fatty Acids
OA/PB	Organic Acid/Pure Botanical
OTUs	Operational Taxonomic Unit
PFTN	Pair-Fed Thermal Neutral
PRLR	Prolactin Receptor
ROS	Reactive Oxygen Species
SCC	Somatic Cell Counts
SCFA	Short-Chain Fatty Acids
THI	Temperature–Humidity Index
